# Cellular mechanisms of monozygotic twinning: clues from assisted reproduction

**DOI:** 10.1093/humupd/dmae022

**Published:** 2024-07-12

**Authors:** Hongbin Jin, Yang Han, Jennifer Zenker

**Affiliations:** Australian Regenerative Medicine Institute, Monash University, Clayton, VIC, Australia; Division of Cellular and Developmental Biology, Molecular and Cell Biology Department, University of California, Berkeley, CA, USA; Australian Regenerative Medicine Institute, Monash University, Clayton, VIC, Australia

**Keywords:** monozygotic twins, assisted reproduction, embryo development, chorion, inner cell mass, blastocyst cavitation, zona pellucida, assisted hatching

## Abstract

**BACKGROUND:**

Monozygotic (MZ) twins are believed to arise from the fission of a single fertilized embryo at different stages. Monochorionic MZ twins, who share one chorion, originate from the splitting of the inner cell mass (ICM) within a single blastocyst. In the classic model for dichorionic MZ twins, the embryo splits before compaction, developing into two blastocysts. However, there are a growing number of ART cases where a single blastocyst transfer results in dichorionic MZ twins, indicating that embryo splitting may occur even after blastocyst formation.

**OBJECTIVE AND RATIONALE:**

For monochorionic MZ twins, we conducted a comprehensive analysis of the cellular mechanisms involved in ICM splitting, drawing from both ART cases and animal experiments. In addition, we critically re-examine the classic early splitting model for dichorionic MZ twins. We explore cellular mechanisms leading to two separated blastocysts in ART, potentially causing dichorionic MZ twins.

**SEARCH METHODS:**

Relevant studies including research articles, reviews, and conference papers were searched in the PubMed database. Cases of MZ twins from IVF clinics were found by using combinations of terms including ‘monozygotic twins’ with ‘IVF case report’, ‘ART’, ‘single embryo transfer’, or ‘dichorionic’. The papers retrieved were categorized based on the implicated mechanisms or as those with unexplained mechanisms. Animal experiments relating to MZ twins were found using ‘mouse embryo monozygotic twins’, ‘mouse 8-shaped hatching’, ‘zebrafish janus mutant’, and ‘nine-banded armadillo embryo’, along with literature collected through day-to-day reading. The search was limited to articles in English, with no restrictions on publication date or species.

**OUTCOMES:**

For monochorionic MZ twins, ART cases and mouse experiments demonstrate evidence that a looser ICM in blastocysts has an increased chance of ICM separation. Physical forces facilitated by blastocoel formation or 8-shaped hatching are exerted on the ICM, resulting in monochorionic MZ twins. For dichorionic MZ twins, the classic model resembles artificial cloning of mouse embryos *in vitro*, requiring strictly controlled splitting forces, re-joining prevention, and proper aggregation, which allows the formation of two separate human blastocysts under physiological circumstances. In contrast, ART procedures involving the transfer of a single blastocysts after atypical hatching or vitrified-warmed cycles might lead to blastocyst separation. Differences in morphology, molecular mechanisms, and timing across various animal model systems for MZ twinning can impede this research field. As discussed in future directions, recent developments of innovative *in vitro* models of human embryos may offer promising avenues for providing fundamental novel insights into the cellular mechanisms of MZ twinning during human embryogenesis.

**WIDER IMPLICATIONS:**

Twin pregnancies pose high risks to both the fetuses and the mother. While single embryo transfer is commonly employed to prevent dizygotic twin pregnancies in ART, it cannot prevent the occurrence of MZ twins. Drawing from our understanding of the cellular mechanisms underlying monochorionic and dichorionic MZ twinning, along with insights into the genetic mechanisms, could enable improved prediction, prevention, and even intervention strategies during ART procedures.

**REGISTRAITON NUMBER:**

N/A.

## Graphical abstract

**Figure:**
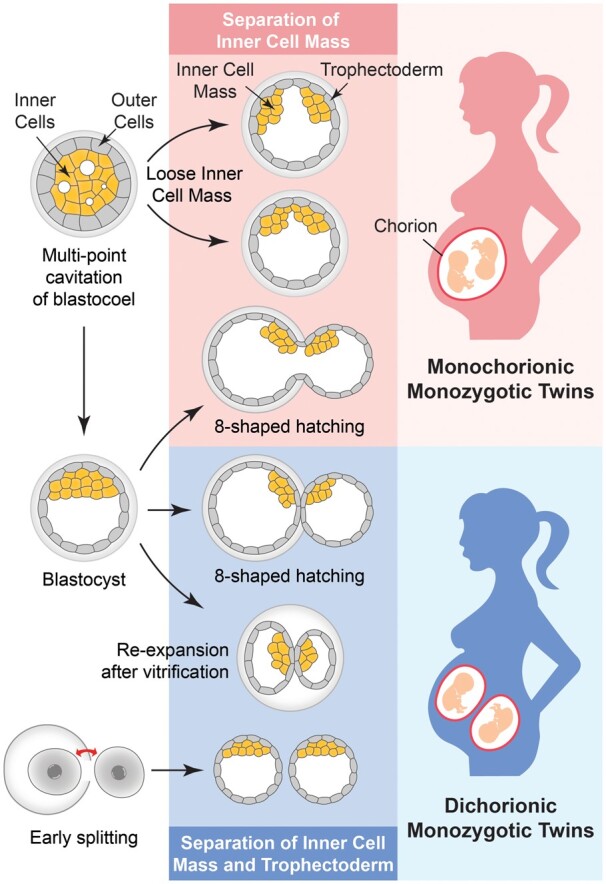
**Splitting of the inner cell mass within a blastocyst leads to monochorionic monozygotic twins; if one embryo splits into two blastocysts, dichorionic monozygotic twins develop**.

## Introduction

In the last four decades, the incidence of twin pregnancies has seen a notable rise worldwide (Imaizumi 2003; Tandberg *et al.* 2007; Martin *et al.* 2012), largely attributed to advancements in ART (Aston *et al.* 2008). Twins can be of two types: dizygotic twins, resulting from the fertilization of two oocytes developing into two distinct embryos (Hoekstra *et al.* 2008), and monozygotic (MZ) twins, which are a natural occurrence of identical individuals originating from a single fertilized embryo (Corner 1955; Hall 2003; McNamara *et al.* 2016). Twin pregnancies pose high risks to both the fetuses and the mother, including twin-to-twin transfusion syndrome, twin reversed arterial perfusion sequence, preterm birth, vanishing twins, and various gestational complications (Pinborg 2005; Corsello and Piro 2010; Waszak *et al.* 2017; Vitucci *et al.* 2020). In some cases, they can even result in the birth of conjoined twins (Johnston 2001; Kaufman 2004). Whereas the incidence of dizygotic twins can be reduced through single embryo transfer (De Sutter 2006), preventing MZ twins remains challenging because the exact cause of MZ twinning is not fully understood.

For over 100 years, there has been a longstanding effort to unravel the mysteries surrounding the formation of MZ twins, dating back to the early last century (Corner 1922). This extensive history has been comprehensively reviewed recently (Herranz 2015). However, the main limitation of MZ twin studies has been the lack of a suitable mammalian model that consistently exhibits a high rate of MZ twin pregnancies, except for the nine-banded armadillo, which remains the only known animal capable of naturally producing identical quadruplets (Enders 2002; Blickstein and Keith 2007). Despite mouse embryos showing some resemblance to human twin development after manual intervention *in vitro* (Landeira *et al.* 2015; Yan *et al.* 2015; Onodera *et al.* 2017), naturally occurring MZ twins in mice are rare and most likely cannot survive to birth probably due to lower cell number compared to normal embryos (McLaren *et al.* 1995). Ethical regulations surrounding human embryo studies further restrict the exploration of MZ twins. Only recently, with the rapid advancements and the widespread use of ART, the real-time observation of human embryos *in vitro* before implantation has become more feasible (Sutherland *et al.* 2019; Sciorio and Meseguer 2021; Matorras *et al.* 2023). The natural rate of MZ twins is about 0.4% (Murphy and Hey 1997), but higher rates of MZ twins have been reported amongst ART cases compared with natural pregnancies, ranging from 0.72% to 5% (Behr *et al.* 2000; Toledo 2005; Vitthala *et al.* 2009; Knopman *et al.* 2010; Papanikolaou *et al.* 2010; Sharara and Abdo 2010; Kawachiya *et al.* 2011; Vela *et al.* 2011; Luke *et al.* 2014; Mateizel *et al.* 2016; Parazzini *et al.* 2016). Thus, studies on MZ twinning during ART provide valuable clues to explore the unknown mechanisms involved (Aston *et al.* 2008).

**Figure 1.**
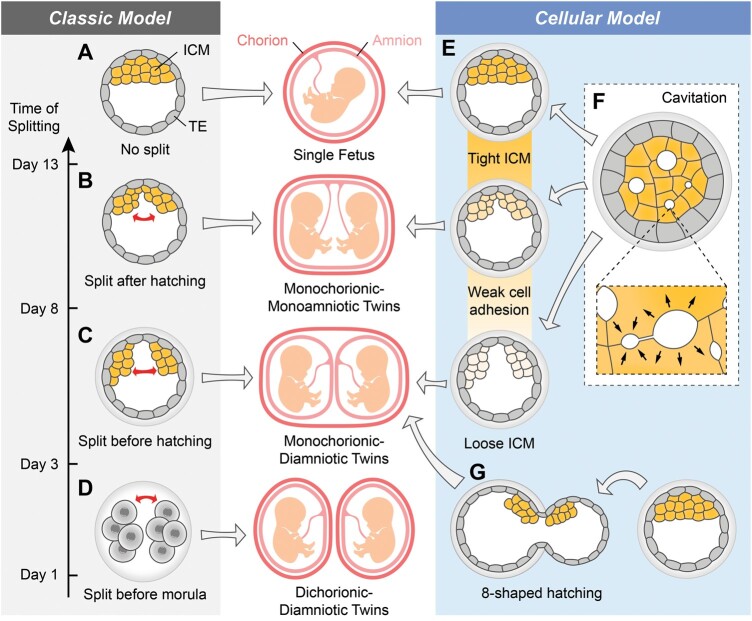
**Classic model for different types of monozygotic (MZ) twinning (left) and cellular model for monochorionic MZ twin formation (right).** (**A**) During human embryo development, the two cell lineages at the blastocyst stage, the inner cell mass (ICM, yellow) and the trophectoderm (TE, gray), primarily develop into the fetus and the chorion, respectively. (**B** and **C**) Monochorionic MZ twins share one chorion, and in most cases, they have their own amnion (third row) but in rare case, they can also share one amnion (second row). Classically, monochorionic MZ twins are formed when the ICM undergoes splitting before or after the blastocyst hatching. (**D**) Dichorionic MZ twins result from early embryo splitting before the morula stage. (**E**) Looseness of the ICM can occur during (**F**) the multi-point initiation of cavitation and there is subsequent accumulation into a single-dominant blastocoel (inset). (**G**) The 8-shaped hatching blastocysts are likely to undergo ICM separation when the ICM is positioned near the hatching point.

MZ twins are classified based on the number of amnion and chorion, which are membranes that surround and protect the fetus during the pregnancy. In humans, the chorion is primarily developed from the trophectoderm (TE), which constitutes the outer cells of a blastocyst, while the pluripotent inner cell mass (ICM) of a blastocyst mainly contributes to all tissues of the fetus (Fig. 1A) (Rossant and Tam 2022). It is widely believed that if the ICM splits into two groups within one blastocoel surrounded by TE at the blastocyst stage or later, it results in monochorionic MZ twins who share one chorion (Fig. 1B and C) (Hall 2003; McNamara *et al.* 2016). On the other hand, dichorionic MZ twins result from the separation of both the ICM and TE. This separation is classically thought to arise from the splitting of blastomeres before the morula stage, during the initial 3 days of human embryo development (Fig. 1D) (Hall 2003; McNamara *et al.* 2016). This leads to the development of two separate blastocysts, giving rise to MZ twins, each with its individual chorion. Recent ART cases, however, diverge from this long-held belief, reporting that a single blastocyst transfer can lead to dichorionic MZ twin pregnancy, which suggests late splitting after the blastocyst stage (Klein *et al.* 2005; Sundaram *et al.* 2018; Dirican and Olgan 2021; Semrl *et al.* 2023).

In this review, we will present evidenced cellular mechanisms of monochorionic and dichorionic MZ twin formation, based on recently reported ART studies as well as mouse embryo research, to propose a new model for the generation of MZ twins, which could potentially contribute to a reduction in the occurrence of MZ twin pregnancies during ART, thus lowering the health risks for both mother and fetus.

## Methods

Cases of MZ twins from IVF clinics were searched in the PubMed database using various combinations of terms, including ‘monozygotic twins’ along with ‘IVF case report’, ‘ART’, ‘single embryo transfer’, ‘assisted hatching’, or ‘dichorionic’. These papers were subsequently categorized through careful reading based on the potential mechanisms implicated in the cases or those with unexplained mechanisms. Additionally, animal experiments related to MZ twins were searched using terms such as ‘mouse embryo monozygotic twins’, ‘mouse 8-shaped hatching’, ‘zebrafish janus mutant’, ‘nine-banded armadillo embryo’, and ‘blastocyst bisection’. Other papers suggesting mechanisms of MZ twins were collected through regular reading of literature.

## Cellular mechanism of monochorionic monozygotic twinning

It is widely accepted that the separation of the ICM at blastocyst stage or after implantation leads to the development of monochorionic MZ twins (Fig. 1B and C). This is supported by documented cases of human blastocysts containing two separate groups of ICMs in a single blastocoel (Meintjes *et al.* 2001; Mio and Maeda 2008; Noli *et al.* 2015), and even triple ICMs (Lee *et al.* 2008), leading to monochorionic twin or triplet pregnancies respectively. Similarly, in mouse studies, blastocysts with double ICMs (Chida 1990; Waheed 2009) or even implanted embryos with two egg cylinder cups (Hsu and Gonda 1980; Waheed 2009) have been observed *in vitro*. To understand the cellular mechanism underlying these processes, the key questions to address are: (i) the character of ICM splitting and (ii) the nature of the physical force responsible for the splitting.

### Looseness of the inner cell mass: reason for splitting

The classic fission model led to speculations that deficient cell adhesion might be the cause, and a potential role of the cell adhesion molecule E-cadherin in the formation of MZ twins was proposed (Bamforth *et al.* 2003). In assisted reproduction, birth rates of MZ twins increased from 0.38% to 1.38% when blastocysts with a poorer grade of ICM quality were transferred, which include those with a looser ICM (Otsuki *et al.* 2016; Eliasen *et al.* 2021).

This theory was supported by a mouse experiment which was not originally designed to study MZ twins but has profound implications (Landeira *et al.* 2015). Mouse embryonic stem cells (mESCs) that lacked *Jarid2* (Jumonji, AT-rich interactive domain 2), a component of the polycomb repressor complex 2, showed a significant reduction in the level of E-cadherin and other genes controlling cell adhesion. Interestingly, when *Jarid2*-null mESCs were injected into the blastocoel of mouse embryos, multiple ICMs were observed in a single blastocyst. Furthermore, the absence of *Jarid2* upregulated ICM-lineage marker *Nanog* and downregulated planar cell polarity signaling genes *Wnt9a*, *Prickle1*, and *Fzd2*. Notably, injection of mESCs overexpressing *Nanog* or mESCs depleted of *Wnt9a*, *Prickle1*, and *Fzd2* also resulted in two or more ICMs in ∼35–48% of blastocysts. Thus, reduced cell adhesion and a looser ICM are most likely significant factors contributing to the splitting of the ICM and consequently might lead to monochorionic MZ twinning (Fig. 1E).

### The physical force that splits the inner cell mass

#### Cavitation of the blastocyst

Extended embryo culture and blastocyst transfer, as compared to the transfer of earlier cleavage-stage embryos, are significant factors contributing to a higher rate of MZ twins during ART procedures (Jain *et al.* 2004; Skiadas *et al.* 2008; Kawachiya *et al.* 2011; Ding *et al.* 2018; Hviid *et al.* 2018; Liu *et al.* 2018a; Busnelli *et al.* 2019). During the formation of the blastocoel before blastocyst transfer in ART, the intermittent collapse and re-expansion process can potentially lead to the separation of the ICM in human embryos (Payne *et al.* 2007; Mio and Maeda 2008). It was further verified in a human *in vitro* model for monochorionic twins that the separation of the ICM can happen during cavitation (Luijkx *et al.* 2024). Thus, the nature of the blastocyst cavitation process may offer valuable insights into the mechanisms underlying the splitting of the ICM, contributing to the occurrence of MZ twins.

While there are no more in-depth studies available on human embryos, research in mice has shown that the initiation of blastocoel formation in mouse preimplantation embryos does not start at a single point but rather begins from hundreds of micrometer-sized lumens formed between cell–cell junctions through hydraulic fracturing (Fig. 1F) (Dumortier *et al.* 2019). These small water-filled pockets gradually release their content, eventually leading to the formation of a single-dominant lumen, the blastocoel. When maternal mutant embryos lacking the cell adhesion molecule *Cadherin 1* (*Cdh1*) were combined with wildtype embryos to form chimera embryos, the final blastocoel lumen was collected alongside the *Cdh1* knockout cells. This suggests that the direction of lumen accumulation tends to separate regions with lower cell–cell contacts.

Altogether, if the inner cells of the preimplantation embryo, undergoing blastocoel formation, are loosely connected, they are more likely to be separated through multipoint cavitation and the accumulation of fluids. This acts as a physical force that splits the ICM into two or three distinct groups in one blastocyst, ultimately resulting in the formation of MZ twins who share a chorion (Fig. 1E and F).

#### 8-Shaped hatching

In ART, assisted hatching is widely used to help an embryo escape from the zona pellucida (ZP), thereby promoting its progression toward implantation. Various techniques of ZP manipulation can assist hatching, including mechanical dissection, drilling, and thinning (Cohen 1991; Hammadeh *et al.* 2011; Schimmel *et al.* 2014). While assisted hatching is not always found to be significantly associated with MZ twinning (Sills *et al.* 2000; Elizur *et al.* 2004; Vitthala *et al.* 2009; Wu *et al.* 2014; Gu *et al.* 2018; Busnelli *et al.* 2019), its potential contribution to MZ twinning during ART procedures is controversially discussed (Saito *et al.* 2000; Alikani *et al.* 2003).

When an artificial small hatching slit is created on the ZP by mechanical assisted hatching or ZP drilling, the blastocyst will hatch through this opening, taking on a shape resembling the number ‘8’ (Fig. 1G). In an IVF case, time-lapse live imaging of an 8-shaped hatching human blastocyst revealed that the ICM situated near the hatching point passed through this hatching hole in the blastocyst and divided into multiple parts (Fig. 1G) (Sutherland *et al.* 2019). One part of the ICM remained inside the ZP, while the other two parts were observed outside, and would thus result in a monochorionic triplet pregnancy. During such 8-shaped hatching events, some of the ICM cells near the hatching point may undergo apoptosis, and the pressure from the narrow gap on the ZP separates the ICM into distinct groups (Ménézo and Sakkas 2002).

Mouse experiments have also provided support for this phenomenon, showing that 8-shaped hatching increases the separation of the ICM at blastocyst stage (Yan *et al.* 2015; Onodera *et al.* 2017). This 8-shaped hatching occurs in over 20% of mouse embryos *in vitro* (Yan *et al.* 2015). The relative position between the hatching point and ICM has been demonstrated to be vital for ICM separation during 8-shaped hatching, similar to human embryos (Onodera *et al.* 2017). If the ICM is located near the hatching point, it is more likely to result in the separation into two or more groups of ICM (Fig. 1G).

However, animal studies have revealed that the hatching process *in vivo* differs significantly from that *in vitro* (Montag *et al.* 2000; Seshagiri *et al.* 2009; Vajta *et al.* 2010). *In vivo*, hatching occurs rapidly with the assistance of lytic factors like proteases present in the uterus. The ZP undergoes global solubilization and complete lysis without expansion and collapse *in vivo*. However 8-shaped hatching is more likely to happen *in vitro* when a small hatching point or gap is created in the ZP by assisted hatching (Yan *et al.* 2015).

### Monochorionic monoamniotic monozygotic twins

In the late human blastocyst, the ICM undergoes differentiation into two distinct cell types: the hypoblast, which gives rise to the yolk sac, and the epiblast, which develops into the embryonic body and the amnion, a membrane that directly surrounds the human fetus (Molè *et al.* 2020; Rossant and Tam 2022). In instances where the epiblast of the ICM is not completely separated into two parts but remains partially connected, the amnion of twins within a single chorion can also merge, leading to monochorionic monoamniotic twins (Fig. 1B). Further investigation is required to identify factors that regulate the spacing between separated ICM clusters during blastocyst cavitation, thereby influencing the number of amnions.

A similar phenomenon can be observed in the zebrafish MZ twin mutant known as *janus*, where the spacing of blastomeres also determines the outcome of the twin phenotype (Abdelilah *et al.* 1994; Abdelilah and Driever 1997). In the Zebrafish *janus* mutant, blastomeres divide into two groups during the first four cleavages and ultimately attach to different sites on the embryonic yolk. If the distance between the separated blastomeres is too close, the blastoderm will partially fuse during development, resulting in a conjoined marginal zone (Abdelilah and Driever 1997). While the *janus* mutant can mimic certain aspects of the ICM separation process, the phenotype is unstable, and the mutated gene has not been identified.

However, according to the classic fission model of human monochorionic monoanionic MZ twins, it is believed that they arise from the division of the ICM subsequent to hatching, even after implantation, occurring after embryonic day 8 (Fig. 1B) (Hall 2003; Kaufman 2004; McNamara *et al.* 2016). It is unclear whether monochorionic monoamniotic MZ twins result from partial ICM splitting during blastocyst cavitation or after hatching. While this type of MZ twins is uncommon for twin pregnancies, accounting for only 1–2% of liveborn MZ twins (Hall 2003), it poses a significant risk of giving rise to conjoined twins if the two clusters of ICMs are not fully separated (Johnston 2001). Due to their low rate of occurrence and late splitting, it is difficult to observe their development *in vitro* through live imaging. Additionally, the lack of a suitable animal model makes it challenging to study the mechanism of monochorionic monoanionic MZ twinning. Resolving this mystery could significantly contribute to preventing the occurrence of conjoined twins, a situation that poses extremely high health risks to the progeny associated with a substantial financial burden on their families.

## Cellular mechanism of dichorionic monozygotic twinning

In natural pregnancies, dichorionic twins are commonly but not necessarily correctly assumed to be dizygotic, originating from two separately fertilized oocytes. Their zygosity can only be confirmed through genetic testing, like DNA fingerprinting (McLaren *et al.* 1995) and short tandem repeat profiling (Brouillet *et al.* 2022; Semrl *et al.* 2023). As a result, studying the mechanisms of dichorionic MZ twinning has been challenging until the reports of numerous ART cases where single blastocyst transfers resulted in dichorionic twin pregnancies (Klein *et al.* 2005; Kyono 2013; Sundaram *et al.* 2018; Konno *et al.* 2020; Li *et al.* 2020; Neumann *et al.* 2020; Dirican and Olgan 2021; Brouillet *et al.* 2022; Semrl *et al.* 2023). Most recently, there has even been the first reported case of a dichorionic diamniotic triplet pregnancy after a single blastocyst transfer (Cara *et al.* 2023). However, these dichorionic twins might have been dizygotic even after single embryo transfer, with one of the twins developing from the transplanted embryo of ART, while the other one developed through natural conception following ovulation (van der Hoorn *et al.* 2011; Osianlis *et al.* 2014; Takehara *et al.* 2014). To eliminate the possibility that these dichorionic twins are dizygotic, some studies have conducted genetic testing to confirm their monozygosity (Krishnan *et al.* 2008; Brouillet *et al.* 2022; Semrl *et al.* 2023).

**Figure 2.**
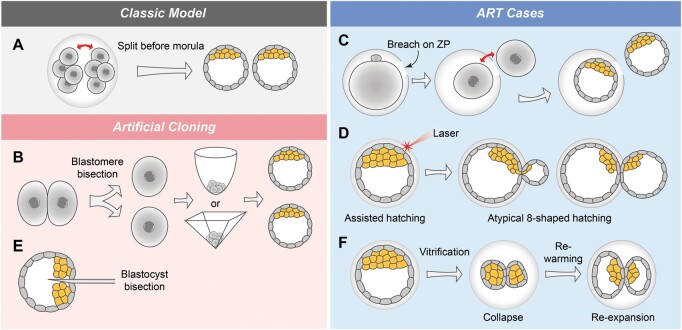
**Formation of dichorionic monozygotic (MZ) twins**. (**A**) The classic model of dichorionic MZ twin formation proposed that it occurs when blastomeres split before the morula stage. (**B**) The divided blastomeres of zona pellucida (ZP)-free mouse embryos at the two-cell stage can be separately cultured in U-shaped or V-shaped bottom wells, eventually developing into small blastocysts. (**C**) In an ART case, one of the blastomere emerged from the ZP through a breach at the two-cell stage, with each blastomere forming an individual blastocyst. (**D**) Dichorionic MZ twins can result from the separation of the ICM and trophectoderm during atypical 8-shaped hatching, forming two individual small blastocysts. (**E**) In certain species such as sheep, cattle, goat, and pig, embryos can be replicated by splitting a blastocyst into two halves using a sharp needle, each containing a similar number of ICM and TE cells. (**F**) Blastocyst separation was observed in an ART case in a vitrified-warmed cycle, leading to a dichorionic MZ twin pregnancy.

Yet, the main point of contention in dichorionic twinning is the timing of the splitting process. Initially, it was hypothesized that the separation occurs at an early stage before embryonic day 3 to generate two separate blastocysts, each forming an individual MZ twin with its own chorion and amnion (Fig. 2A) (Corner 1922). Later it was proposed that the splitting can occur even as early as right after the first cleavage at two-cell stage, between the two blastomeres (Herranz 2015). However, the recent ART cases suggest that the splitting of dichorionic MZ twins may occur at the blastocyst stage or later, which could represent an alternative mechanism compared to the existing model (Klein *et al.* 2005; Kyono 2013; Sundaram *et al.* 2018; Konno *et al.* 2020; Li *et al.* 2020; Neumann *et al.* 2020; Dirican and Olgan 2021; Brouillet *et al.* 2022; Semrl *et al.* 2023). In this section, we will first discuss the classic scenario of early embryo splitting before the morula stage, and then explore the mechanisms behind a single blastocyst transfer leading to dichorionic MZ twins, based on ART cases and mouse experiments.

### Can early splitting lead to dichorionic twins under physiological conditions? Lessons from artificial cloning

Blastomere biopsy of human embryos before compaction allows each group of blastomeres to develop into individual blastocysts (Illmensee *et al.* 2010; Noli *et al.* 2016), resembling the classic model of dichorionic MZ twins. However, the occurrence of this process under physiological conditions without manual intervention remains controversial.

Generation of MZ twins through blastomere separation was first performed in sheep and cow embryos (Willadsen 1980, 1989; Willadsen and Polge 1981). In mouse embryos, recent findings suggest that cell fate has diverged between the cells as early as the two-cell stage, with one blastomere exhibiting stronger totipotency than the other (Papaioannou *et al.* 1989; Casser *et al.* 2017; Hupalowska *et al.* 2018; Wang *et al.* 2018; Jin *et al.* 2022). However, in some cases, it is still possible to obtain artificially generated twin blastocysts and individuals through bisecting the blastomeres at the two-cell stage (Fig. 2B) (O'Brien *et al.* 1984; Motosugi *et al.* 2005; Roberts *et al.* 2011; Morris *et al.* 2012; Hisaki *et al.* 2014; Casser *et al.* 2017, 2019; Krawczyk *et al.* 2021). These artificial cloning processes could provide us with a valuable indication of the feasibility of the early splitting theory of dichorionic MZ twins.

Strict conditions are required to successfully generate MZ twin embryos in mice through blastomere separation. Firstly, the embryo needs to be released from the ZP by either creating a slit in the ZP or by completely removing the ZP with Tyrode's solution. Then, the sister blastomeres are to be separated using physical force like pipetting or a chemical agent like Trypsin. Next, to prevent conjoining, the ZP-free half embryos are to be cultured in separated spaces to block any junction between them. Furthermore, to ensure the proper 3-dimensional aggregation of blastomeres into blastocysts without the presence of the ZP, the bisected blastomeres are to be cultured in a restricted environment such as U-shaped or V-shaped bottom wells (Fig. 2B) (Casser *et al.* 2017; Liu *et al.* 2021; Yu *et al.* 2021), or even placed back into empty ZPs (Illmensee *et al.* 2005; Motosugi *et al.* 2005; Tang *et al.* 2012).

Drawing from the evidence of mouse experiments (Motosugi *et al.* 2005; Tang *et al.* 2012; Casser *et al.* 2017), three key questions need to be addressed on how separated human blastocysts can be achieved from early splitting embryos without manual intervention and under physiological conditions as hypothesized by the classic theory.

#### What physical force causes the splitting of blastomeres before the morula stage?

A thin ZP or a breached ZP is associated with MZ twinning, where the embryo can be relieved from the restriction of the ZP and undergo splitting (Alikani *et al.* 1994). Recently, an ART case of a spontaneous early splitting human embryo was observed using time-lapse live imaging (Matorras *et al.* 2023). The ZP of the early splitting human embryo ruptured during oocyte manipulation (Fig. 2C). Following sperm microinjection and the first cleavage, one of the two blastomeres emerged from the ZP, while the other remained inside. The two separated blastomeres developed individually into blastocysts, having the potential to give rise to dichorionic MZ twins (Fig. 2C). However, to make this scenario happen under physiological conditions, the question of which forces or factors can cause the rupture of the ZP *in vivo* needs to be answered.

The early splitting of the embryo could result from multiple mechanisms. An interphase bridge, a microtubule cytoskeleton-dense structure connecting sister blastomeres during interphase within the preimplantation embryo (Zenker *et al.* 2017), might lead to the separation of cells when breaking down during cell division. Additionally, the repulsion and contact inhibition could potentially occur between two separate blastomeres, mediated by pathways such as Eph/ephrin signaling, which regulates cell–cell contact and repulsion to preserve cellular or tissue boundaries (Pasquale 2005; Klein 2012; Zhang *et al.* 2016). A decrease in calcium levels has also been hypothesized to increase the rate of MZ twins by regulating cell adhesion and inducing early embryo splitting (Steinman 2001a,b; Steinman and Valderrama 2001).

#### How do twin embryos prevent themselves from rejoining in a physiological environment?

Ensuring the separation of blastocysts is crucial to avoid the fusion of embryos, as human embryos can fuse in group cultures (Schiewe *et al.* 2015; Swain 2021). In some cases, the chorions of dizygotic twins can fuse into one (Peters *et al.* 2017). Similarly, mouse blastocysts can also be fused into one chimeric blastocyst with two clusters of ICMs (Tarkowski and Wojewodzka 1982). Zebrafish MZ twins maintain their separation before hatching by attaching to different sites of the yolk, a unique feature that mammalian embryos lack (Abdelilah *et al.* 1994; Abdelilah and Driever 1997). Thus, to support the early-splitting theory of dichorionic MZ twins under a physiological environment, factors or mechanisms which could keep the twin blastomeres apart until implantation occurs need to be further investigated. One possible scenario could involve a breached-ZP, with one twin embryo developing outside, and the other inside, the ZP (Fig. 2C) (Matorras *et al.* 2023). This could also potentially be attributed to the cilia-driven fluid flow in the oviduct (Huang and Choma 2015), creating a spatial gap between the two distinct embryos.

#### How do the twin blastomeres ensure proper aggregation to avoid blastomere dispersal without ZP?

Women carrying a mutation in a ZP gene (*ZP1*, *ZP2*, or *ZP3*) face challenges in conceiving naturally due to the risk of abnormal fertilization and improper preimplantation embryo aggregation (Huang *et al.* 2014; Chen *et al.* 2017; Zhou *et al.* 2019, 2022; Luo *et al.* 2020; Sun *et al.* 2021; Zeng *et al.* 2023). ARTs such as ICSI and *in vitro* culture can help address these issues. In most cases, to ensure an intact embryo and successful pregnancy, ZP-free human embryos are cultured *in vitro* until the blastocyst stage before transplantation (Ueno *et al.* 2014; Dai *et al.* 2019; Cao *et al.* 2020; Watson *et al.* 2021). The earliest successful time point for ZP-free embryo transfer is Day 3 when the embryos begin to compact, which can improve the rate of successful pregnancy (Mansour *et al.* 2000). To enhance the development rate of ZP-free human embryos, the Well-of-the-Well (WOW) system was applied *in vitro* where V-shaped small wells are created within a larger well to facilitate the proper compaction (Vajta *et al.* 2000, 2008). ZP-free human embryos can also be placed back into empty ZP (Illmensee *et al.* 2010) or an artificial gel to support the development of these embryos (Song *et al.* 2022).

However, under physiological conditions, if the embryo split before morula stage, ZP-free twin embryos are challenging to maintain in an intact state due to low cell adhesion (Fleming *et al.* 2001), and may experience difficulties in undergoing normal compaction and blastocyst formation. To address whether early-split, ZP-free twin embryos can achieve proper aggregation and successful embryo development *in vivo*, we need model systems mimicking the physiological conditions. Several experimental setups have been established to replicate physiological fluid flow *in vitro* (Juste-Lanas *et al.* 2023), which can simulate the oviduct environment and study the development of early ZP-free embryos.

Overall, to generate dichorionic MZ twins through early splitting of embryos in physiological condition, the embryo must satisfy several stringent criteria: they require a physical force to split and exit the ZP at an early stage, while developing intact without dispersing after splitting, and they must also avoid touching and fusing with each other. The occurrence of dichorionic MZ twinning through early splitting is potentially lower under physiological conditions (Gardner 2014). Further studies and supporting evidence from both ART cases and animal experiments are necessary to understand how dichorionic MZ twins happen under physiological conditions.

### Mechanisms of dichorionic monozygotic twinning after single blastocyst transfer

#### Atypical hatching causes higher rates of dichorionic MZ twins in ART

In ART, several cases have been reported where atypical hatching has led to a dichorionic MZ twin pregnancy after single blastocyst transfer (Van Langendonckt *et al.* 2000; Behr and Milki 2003; Kyono 2013; Sundaram *et al.* 2018; Jundi *et al.* 2021). In some atypical cases of the 8-shaped hatching, the slit on the ZP is small, allowing only half of the ICM to emerge from the ZP, while the other half remains inside. When both, the ICM and the TE, divide into two halves due to the pressure from the small slit drilled by laser, it may result in the formation of two separated blastocysts, leading to MZ twins, each with their own chorion (Fig. 2D) (Van Langendonckt *et al.* 2000; Sundaram *et al.* 2018; Jundi *et al.* 2021). This concept bears resemblance to the method of artificial cloning used in animal husbandry for mammals such as sheep, cattle goats, and pigs, where the blastocyst is directly bisected into two halves using a sharp needle (Fig. 2E) (Willadsen and Godke 1984; Williams *et al.* 1984; Tsunoda *et al.* 1985; Nagashima *et al.* 1989; Széll and Hudson 1991; Noli *et al.* 2016).

#### Vitrified-warmed cycles can lead to dichorionic MZ twins

To date, there is only one reported case in which an embryo from a vitrified-warmed cycle was observed to separate into two blastocysts, leading to the birth of dichorionic MZ twins (Fig. 2F) (Shibuya and Kyono 2012). During the re-expansion of the blastocoel after embryo freezing and re-warming, some cells may remain intact, coincidentally segregating the ICM and TE into two small, separated blastocysts formed inside the ZP (Fig. 2F). While this is the only reported case with a clear phenotype of blastocyst segregation, there are also unexplained cases of dichorionic MZ twin pregnancies following the transfer of a single vitrified-warmed blastocyst, which might share the same underlying mechanism (Li *et al.* 2020; Semrl *et al.* 2023).

## Limitations and direction for future studies

### Current models of monozygotic twinning and their limitations

Although humans are not the only species that exhibits MZ twins, there is currently no perfect animal model available to study the mechanism of human MZ twinning, which hinders research in this area. In this section, we will summarize the species currently known for MZ twinning and explain their limitations for studying human MZ twinning. Furthermore, we will propose *in vitro* human embryo models that hold promise for studying human MZ twins in future research.

#### Zebrafish

**Figure 3.**
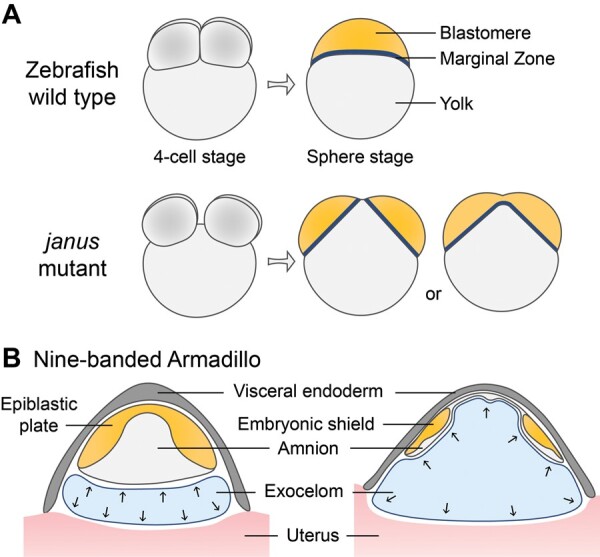
**Animals that can produce monozygotic (MZ) twins or quadruplets**. (**A**) The zebrafish *janus* mutant exhibits the separation of two groups of blastomeres prior to the eight-cell stage, resulting in the development of two distinct spheres (yellow) that ultimately lead to the formation of conjoined fish. If there is limited space between the two groups of cells, the blastomeres and marginal zones (dark blue) will merge during development. (**B**) To generate MZ quadruplets, zygotic splitting of nine-banded armadillo embryos happens after implantation. The substantial enlargement of the exocelom cavity (blue) functions as a physical force, causing the division of the embryonic shields that develop from the epiblastic plate (yellow), and effectively separating them into distinct spaces that remain unconnected.

Zebrafish is a widely used vertebrate model organism, leveraging its significant advantage of *in vitro* fertilization and growth, which allows for the comprehensive observation of the entire developmental process (Astell and Sieger 2020). However, it exhibits notable differences in embryo structure and developmental patterns compared to mammalian embryos (upper panel of Fig. 3A), posing challenges in its application for studying human twinning. A zebrafish MZ twin mutant, known as *janus*, displays two distinct clusters of blastomeres attached closely to a single yolk (lower panel of Fig. 3A) (Abdelilah *et al.* 1994). However, the mechanisms governing the splitting of blastomeres are not fully understood, and the gene responsible for this natural mutation has yet to be identified. The primary challenge is the instability of the phenotype, which is temperature-sensitive and inherited through a maternal recessive mode (Abdelilah and Driever 1997). Overall, the structural disparities of zebrafish embryos and intricate inheritance patterns of the mutant render it less than an ideal animal model for studying human MZ twinning.

#### Nine-banded armadillo

The nine-banded armadillo is the sole animal known to naturally and consistently give birth to identical quadruplets (Carter 2018). However, the timing of zygotic splitting occurs after implantation, which is markedly distinct from human twinning (Prodöhl *et al.* 1996; Enders 2002; Blickstein and Keith 2007). In the nine-banded armadillo, the embryo does not implant right after the formation of blastocyst. Instead, it experiences a delay that can span several months. After blastocyst implantation, the ICM gives rise to a single amnion and an epiblastic plate. Between the TE implantation site and the amnion, a distinctive cavity named the exocelom forms (Fig. 3B) (Enders 2002). Subsequently, the epiblastic plate undergoes differentiation into separated embryonic shields, each capable of developing into an individual. As the exocelom cavity expands significantly, it works as a physical force and ultimately splits the four embryonic shields to distinct locations, giving rise to identical quadruplets (Fig. 3B). This mechanism is unique to this specific species and does not offer significant insights into human MZ twinning. Additionally, the nine-banded armadillo is not commonly employed as a model organism used in research laboratories and is thus not readily accessible for embryonic research purposes.

#### Mouse

While mice are the most commonly used mammalian model in current scientific studies, fundamental differences confine their resemblance with human embryos for MZ twin research. The natural occurrence of mouse MZ twinning under physiological conditions is exceedingly rare (McLaren *et al.* 1995). One significant issue is that mice are not a single-birth animals, making it challenging to identify which two embryos originated from one oocyte without genetic testing. Additionally, murine MZ twin embryos might thus face disadvantages by having fewer cells than the neighboring embryos that originate entirely from a single fertilized oocyte, which could result in their loss during natural competition. Therefore, it is less likely to obtain MZ twins in mice. Moreover, the development of mouse embryos differs significantly from that of human embryos in various aspects, such as timing of zygotic genome activation, compaction, implantation, gastrulation, and more (Molè *et al.* 2020; Bissiere *et al.* 2023). Especially, human embryos implant from the polar TE attaching to the ICM, while mouse embryos implant from the mural TE which is opposite to the ICM (Muter *et al.* 2023). Due to these differences, the development of MZ twins cannot be fully replicated or accurately mimicked using mouse embryos, which possess their unique characteristics. It is only useful in situations where insights from human ART cases were obtained and potential mechanisms have been evaluated and validated in mouse embryos at specific preimplantation stages, such as atypical 8-shaped hatching and ICM separation. Thus, studying MZ twinning in human embryos during ART procedures with increased MZ twin birth rates still offers the most direct and effective approach to address the fundamental questions raised in this review.

#### In vitro models of human embryogenesis

In ART therapy, human embryos can only be cultured *in vitro* to blastocyst stage before they are implanted into the uterus, which is a main restriction in research to observe the human embryo beyond the blastocyst stage. The extended cultivation of human embryos *in vitro* for scientific research is strictly limited due to ethical considerations. As a result, we are lacking information about whether the ICM can divide after hatching or even after implantation to generate monochorionic MZ twins. Furthermore, there have been numerous inexplicable ART cases where a single blastocyst transfer leads to MZ twins (Konno *et al.* 2020; Li *et al.* 2020; Neumann *et al.* 2020; Semrl *et al.* 2023) or even MZ triplets (Faraj *et al.* 2008; Lee *et al.* 2008; Dessolle *et al.* 2010; Gurunath *et al.* 2015; Saravelos *et al.* 2016). Recent advancements in creating *in vitro* systems to model early human embryogenesis using induced pluripotent stem cells (iPSCs) or isolated human embryonic stem cells have shown promising results, with the development of blastocyst-like structures called blastoids (Liu *et al.* 2021; Yu *et al.* 2021) and even post-implantation human embryoids (Pedroza *et al.* 2023; Weatherbee *et al.* 2023). These models may provide unprecedent opportunities to investigate whether the ICM and TE can still separate after hatching or even after implantation.

In fact, double ICMs within a single blastocoel have been observed in some twin blastoids, a valuable model for studying human MZ twins *in vitro*, by increasing the cell number and treating the twin blastoids with lysophosphatidic acid. The ICMs separated during cavity expansion, with each ICM containing both epiblast and hypoblast cells, mimicking the phenotype of monochorionic MZ twins. This *in vitro* model system may be further used to investigate the mechanisms underlying ICM separation, the control of ICM spacing, and post-implantation development of MZ twins (Luijkx *et al.* 2024).

### Searching for ‘twin genes’

Currently, our understanding of the mechanism of MZ twins is primarily centered around the cellular level, and the genetic mechanisms that drive this process still require further investigation and discovery. There is a contention that the elevated rate of MZ twins in ART is not attributed to the technology itself, but rather to the genetic background of the embryos (Sobek *et al.* 2015).

Scientists believe that MZ twins are controlled by genetic regulation for two reasons. Firstly, unlike dizygotic twins, the occurrence of MZ twins within the population is relatively consistent across different regions, with an approximate rate of 1 in 250 pregnancies (McNamara *et al.* 2016). Secondly, there have been reports of familial cases of MZ twins spanning up to four generations (Steinman 2003; Hamamy *et al.* 2004; Cyranoski 2009; Machin 2009a; Liu *et al.* 2018b). Numerous studies have been conducted by scientists to uncover the genetic mechanisms underlying the formation of MZ twins (Lichtenstein *et al.* 1998; Cyranoski 2009; Liu *et al.* 2018b). MZ twinning is inherited in an autosomal dominant manner, and genes related to cell adhesion are consistently on the list of suspects responsible for MZ twinning (Bamforth *et al.* 2003; Machin 2009b). Through whole genome sequencing of a four-generation MZ twin pedigree, enrichment of single-nucleotide variants and copy number variants were observed within the epithelial adherens junction signaling pathway, GTPase family-mediated pathways, and tight junction signaling pathways (Liu *et al.* 2018b). Mutated genes may reduce the adhesion of the ICM, resulting in the division of the ICM during the cavitation of the blastocoel. Despite significant efforts, specific genes directly responsible for human MZ twins have not yet been identified. The genetic mechanism behind MZ twins is challenging to uncover because it may not be linked to the mutation of a single gene, and even if there is a mutation, its effect may not be fully penetrant.

In addition to the genetic mechanism, epigenetic hallmarks for MZ twins have recently been identified (van Dongen *et al.* 2021, 2024). Differentially methylated positions between MZ and dizygotic twins remain consistently present in their somatic cells, gauging a new way of identifying individuals as MZ twins. Genes related to cell-adhesion pathways showed significant enrichment among the genes nearest to the differentially methylated positions.

The discovery of ‘twin genes’ may allow further insights into the cellular mechanism of MZ twins at both the genetic and the epigenetic levels, and into twin rates associated with regional origins or pedigrees.

### Implications for reducing the monozygotic twinning rate in ART therapy

As ART advances and achieves higher success rates, the practice of single embryo transfer has become prevalent to mitigate the occurrence of multiple pregnancies (Pinborg 2005; Reimundo *et al.* 2021; De Neubourg *et al.* 2022; Fouks and Yogev 2022). However, MZ twinning still remains possible following single embryo transfer. Based on the cellular mechanisms analyzed in our review through reported ART cases and animal studies, the following measures should be considered to minimize the occurrence of MZ twins during IVF process. It is essential to thoroughly monitor the development of ART embryos and confirm the phenotype of blastocysts prior to transplantation, using cutting-edge technologies such as high-resolution time-lapse live imaging (Zenker *et al.* 2017, 2018; Sutherland *et al.* 2019; Hawdon *et al.* 2023; Matorras *et al.* 2023). To reduce the occurrence of monochorionic MZ twinning, blastocysts displaying a loosely connected ICM or those divided into multiple groups should be avoided. To decrease the incidence of dichorionic MZ twin pregnancies, embryos displaying atypical 8-shaped hatching or divided blastocysts should not be the primary choice for transplantation. When performing assisted hatching, it is important to create the artificial hatching site at a distance from the ICM to prevent the ICM splitting during 8-shaped hatching. Furthermore, a technique more akin to the natural degradation of the ZP rather than creating a single small hole may be beneficial. Implementing these measures can help to reduce the occurrence of MZ twins in ART to some extent.

## Conclusion

In summary, our knowledge on the cellular mechanisms of monochorionic MZ twins is advancing, and involves loose ICM splitting during multi-point blastocoel expansion, resulting in separate ICM clusters within the blastocyst. On the other hand, the natural occurrence of dichorionic MZ twins remains poorly understood and highly controversial. In ART, atypical 8-shaped hatching and vitrified-warmed cycles have been associated with blastocyst separation and the formation of dichorionic MZ twins. However, the mechanisms occurring under natural physiological conditions appear to be distinct and unclear. To gain further insights, the MZ twinning model requires continuous examination and potential modifications, particularly with the accumulation of future reported cases from ART procedures, animal experiments, and human models using iPSC-derived blastoids, gastruloids, and other embryonic organoids. With updates from MZ twinning models, the embryo transfer strategy in ART should also be adjusted to lower the rate of MZ twins. Additionally, exploring the genetic and epigenetic mechanisms involved will take us another step closer to unraveling the mystery of MZ twins.

## Data Availability

No new data were generated or analyzed in support of this review.
